# Left Ventricular Responses during Exercise in Highly Trained Youth Athletes: Echocardiographic Insights on Function and Adaptation

**DOI:** 10.3390/jcdd9120438

**Published:** 2022-12-06

**Authors:** Viswanath B. Unnithan, Alexander Beaumont, Thomas Rowland, Keith George, Nicholas Sculthorpe, Rachel N. Lord, Andisheh Bakhshi, David Oxborough

**Affiliations:** 1 Sport and Physical Activity Research Institute, Division of Sport and Exercise, School of Health and Life Sciences, University of the West of Scotland, Hamilton G72 0LH, UK; 2 School of Science, Technology and Health, York St. John University, York YO31 7EX, UK; 3 Research Institute for Sport and Exercise Sciences, Liverpool John Moores University, Liverpool L3 3AF, UK; 4 Cardiff Centre for Exercise and Health, Cardiff Metropolitan University, Cardiff CF5 2YB, UK; 5 Phastar, Glasgow, Scotland, UK

**Keywords:** in-exercise, echocardiography, highly trained, youth athletes

## Abstract

There is an increase in the prevalence of elite youth sports academies, whose sole aim is to develop future elite athletes. This involves the exposure of the child and adolescent athlete to high-volume training during a period of volatile growth. The large amount of data in this area has been garnered from the resting echocardiographic left ventricular (LV) evaluation of the youth athlete; while this can provide some insight on the functional adaptations to training, it is unable to elucidate a comprehensive overview of the function of the youth athletes’ LV during exercise. Consequently, there is a need to interrogate the LV responses in-exercise. This review outlines the feasibility and functional insight of capturing global indices of LV function (Stroke Index-SVIndex and Cardiac Index-QIndex), systolic and diastolic markers, and cardiac strain during submaximal and maximal exercise. Larger SVI and QI were noted in these highly trained young athletes compared to recreationally active peers during submaximal and maximal exercise. The mechanistic insights suggest that there are minimal functional systolic adaptions during exercise compared to their recreationally active peers. Diastolic function was superior during exercise in these young athletes, and this appears to be underpinned by enhanced determinants of pre-load.

## 1. Introduction

Across all individual and team sports, there is an increase in the prevalence of elite youth sports academies whose sole aim is to develop future elite athletes. This process involves the exposure of the child and adolescent athlete to both high-volume and intensity training during a period of volatile growth [1]. For most youth sports, establishing a strong aerobic base is important for sporting success [2]. Consequently, evaluating one of the key determinants of this parameter-cardiac structure and function has important performance and health implications.

There are a plethora of studies evaluating the athlete’s heart in the youth athlete [3], and McClean’s et al. [3] meta-analysis of this topic revealed an array of structural and functional adaptations in response to training in the pre- and adolescent athlete. While the evidence from these studies do provide some insight on cardiac structure and function in this young athletic population, it is limited in one respect. Namely, the data is acquired at rest, where the functional manifestations of their unique cardiac physiology are never truly revealed. Therefore, creating an echocardiographic evaluation model that allows an interrogation of cardiac function when the young athlete is exposed to an exercise intensity similar to that seen in their sport appears to be an important adjunct to the resting evaluation of the young athlete. Furthermore, exercise perturbation with associated echocardiographic evaluation may also provide pre-clinical and clinical markers of cardiac pathology unable to be detected at rest in the young athlete. In the adult population, exercise stress echocardiography has demonstrated the capacity to discriminate between functional adaptations to training and myocardial damage [4,5]. There is also limited data to suggest that it is capable of delineating pathology, not seen at rest in paediatric and congenital heart disease patients [6,7].

Consequently, the primary three aims of this narrative review are (1) to provide a brief overview of the structural and functional evidence of the athlete’s heart in the pre-and adolescent athlete; (2) to consider methodological issues associated with the in-exercise echocardiographic acquisition with respect to 2D transthoracic and cardiac strain (ε) imaging; (3) to demonstrate how the in-exercise, echocardiographic-derived insights from the submaximal and maximal evaluation of the elite youth athlete <18 years of age can elucidate the mechanisms that underpin their superior athletic performance. Additionally, propositions for future research in this under-represented, unique youth population and area of exercise cardiac physiology will also be highlighted. We acquired relevant research articles based on the authors’ knowledge of the topic area, in addition to research list scrutiny of known articles.

## 2. Paediatric Athletic Heart at Rest

The influence of exercise training and intensive physical activity on the paediatric athlete’s heart at rest has recently and extensively been reviewed through meta-analytical synthesis [3]. The authors reported larger left ventricular (LV) dimensions (diameter, wall thickness, and mass), yet similar systolic and diastolic functions compared with non-athletic counterparts, irrespective of age. The former findings are concomitant with the adult athlete’s heart literature, particularly pertaining to eccentric hypertrophy observed in those engaged in high dynamic activities [8,9]. Moreover, youth athletes have demonstrated morphological remodelling of the right ventricle (RV) [10,11] and atrial chambers, in comparison to untrained controls [3], thus suggesting global structural adaptations within the youth athlete’s heart. While the meta-analysis in 2018 [3] highlighted training related adaptations, the markers of function were limited to conventionally derived indices. Since then, more studies have been published having reported cardiac (ε) imaging via speckle-tracking echocardiography (STE) owing to technological advancements and implementation of both the right and left ventricles (RV and LV, respectively).

Left ventricular STE provides measures of deformation [12] around multiple planes of motion throughout phases of systole and diastole, and can be categorised through ε, rotations, twisting and their respective rates of motion. These markers, in specific relation to systole, provide additional insight regarding the contractility of the myocardium during ejection, as opposed to the conventionally used ejection fraction (EF) with longitudinal ε being the most reported parameter. Few studies have explored circumferential and twist markers of LV function through STE, although greater LV apical rotation, twist, and circumferential ε at the base and mid-papillary level in elite pre-adolescent soccer players than in controls has recently been reported [13].

Assessment of longitudinal ε of the LV has produced variable findings in terms of the athlete-control inter-group differences in youth athletes at rest. Similar peak global LV [13] and RV [10] ε in pre-adolescent elite soccer players compared with matched controls has recently been reported. However, when comparing controls and youth athletes, others have reported either unremarkable differences in LV longitudinal ε in athletes [14,15,16], higher ε in endurance runners and wrestlers [17], or lower ε in basketballers [15] and soccer players [18]. Beyond global assessment of ε, we [10] and others [18] have also noted lower regional LV and RV ε, respectively, in soccer players than in controls. Normative values of two-dimensional (2D) echocardiographic derived LV longitudinal ε in children has been reported to be approximately −20.2% (95% CI −19.5% to −20.8%) through meta-analysis of 2325 children [19]. Of those studies reporting lower ε in athletes than in controls, the significance of such reductions, especially in the context of normative values, warrants scrutiny. Indeed, Binnetoglu et al. [14] explored ε in different LV geometries, identifying lower 4-chamber longitudinal ε in those classified with concentric remodelling compared with eccentric hypertrophy. Moreover, the physiological importance of lower ε in athletes requires investigation to determine potential maladaptation or potential functional reserve, which can be realised through in-exercise echocardiographic examination. To determine the investigative potential of the in-exercise protocol, the methodological issues related to this technique have to be scrutinised.

## 3. Methodological Considerations of Echocardiography during Exercise

Exercise is the recommended method for eliciting a physiological stress to assess cardiac performance [20]. While this provides a natural and appropriate means for cardiac assessment during heightened cardiovascular demand, the technique of exercise echocardiography is not without challenges. Thus, considerations for accurate and reproducible image acquisition are needed to facilitate the feasibility of in-exercise assessment.

Despite the challenges of conducting stress echocardiography and obtaining optimal images, some studies have previously conducted pre-assessment screening before study enrolment [21] to improve the probability of collecting high quality images. Still, the difficulties with image acquisition during stress echocardiography are exacerbated with tachycardia, noise artifacts, and through greater myocardial movement during exercise, which makes obtaining a consistent imaging plane challenging. Thus, it is pertinent that articles report the reproducibility of data collected during exercise, and not only at rest. Standardising image acquisition is important to obtain consistent images [21] and ideally assessed by the same sonographer to limit inter-observer variabilities. Indeed, test-retest reliabilities of tissue Doppler imaging (TDI) derived LV systolic function (*S*′) and mitral inflow velocity (*E*) were determined during submaximal exercise by Unnithan et al. [1], with coefficient of variations (CoV) ranging between 4.7 and 8.8% and between 3.6 and 5.1%, respectively. Additionally, the same authors also reported CoV for stroke volume (SV), derived as the product of velocity time integral (VTI) from the suprasternal notch and LV outflow tract cross-sectional area, during submaximal exercise (20–60 W) as 4.7–8.8% [1]. Moreover, the reproducibility of Doppler-derived stroke volume in children progressively improved from low-intensity (7.5%), high-intensity (5.2%), and at maximal exercise (4.9%) [22]. Interestingly, despite high physiological demand, reproducibility was better than at rest (6.9%). Others have also reported excellent reproducibility of *E* at peak exercise of 2.8% test-retest coefficient of variation [23]. These data do provide valuable insight to suggest that in-exercise echocardiography is both feasible and reproducible in adolescents from low intensity to maximal exercise. Nonetheless, 2D TDI is limited due to angle dependence and restrictions for TDI to detect parasternal short-axis movement in myocardial segments not aligned parallel to the ultrasound beam [24]. The use of STE may overcome some of these limitations, however, by being relatively angle independent with the capability of assessing motion within multiple directions [24].

Regarding specific studies in paediatric athletes having reported reproducibility of STE during exercise, Unnithan et al. [25] reported similar CoV at 40 W compared with rest for longitudinal (8.2%), circumferential (10.4%), and radial ε (16.7%) [25]. However, as external power output increased to 80 W, CoV increased for all variables (13.8, 12.1 and 20.4%, respectively). With increased body movement during exercise, reverberations and shadowing may influence the quality of speckle tracking, which may lead to underestimation of peak values [24]. Indeed, larger variability at higher intensities is matched with a greater loss of sufficient speckle tracking, with increased cardiovascular demand, thus required heart rate in children [26]. Although Liu et al. [27] identified similar longitudinal and circumferential ε acquired above or below 60 Hz at 160 bpm in children, the quantity of available data at high heart rates (~160 bpm) has been reported to be 59% [27] and 60% [26] for 4-chamber images. Successful speckle tracking relies on sufficient temporal resolution [24], which can be maximised to improve tracking success through elevated frame rates; however, this is at the detrimental limitation of spatial resolution. Moreover, spatial resolution is poorer in the lateral direction; thus, for the greatest possibility of capturing high quality images suitable for offline analysis, studies may wish to focus on ε assessment in the direction of the ultrasound beam [28] (i.e., longitudinal), as a priority. Additionally, studies could consider applying a cubic spline interpolation at high heart rates to limit data loss when assessing LV mechanics throughout the cardiac cycle [21,29], although this is only applicable if the correct peak value is present.

Reduced image quality during physiological stress due to tachypnea and greater chest wall movement may require end-expiration breath-hold during acquisition [30]. However, this places a greater demand for participants to modulate breathing while exercising. This may be partially overcome by acquiring images over a several cycles, but with the trade-off of increased scanning time. Additionally, the choice of body position may influence the cycling efficiency and cardiometabolic responses to upright and semi-supine exercise, with or without a left-lateral tilt [31]. Indeed, variations in success with image acquisition and analysis may be due to the adopted cycling positions. In the adolescent literature, various cycling body positions for stress echocardiography have been used, including upright [23,25,32,33], semi-supine [26], and supine [27]. Irrespective of the mode used, it is important that consideration is given to the knowledge that altering posture will induce different physiological responses; thus, a trade-off exists among standardization, image quality [31], and ecological validity. Nonetheless, while cycling (supine, upright, or semi-supine) offers the opportunity for assessment during exercise [20], these body positions may not appropriately reflect the sporting requirements in athletic populations, consequent to differences in cardiac loading conditions. However, there are no clear recommendations, and body positioning should potentially be applied based on local provision and individual participant needs.

## 4. In-Exercise Echocardiographic Evaluation of the Youth Athlete: Insights from Submaximal Exercise Intensity Testing

The rationale for the evaluation of the youth athlete during submaximal exercise protocols lies with its applicability to elucidate cardiac function at an exercise intensity that mimics the primary intensity domain that most youth athletes engage in for the longest duration, irrespective of their sport. A paucity of studies exist in this area and the following narrative outlines the key findings from this limited body of work.

**Figure 1:**
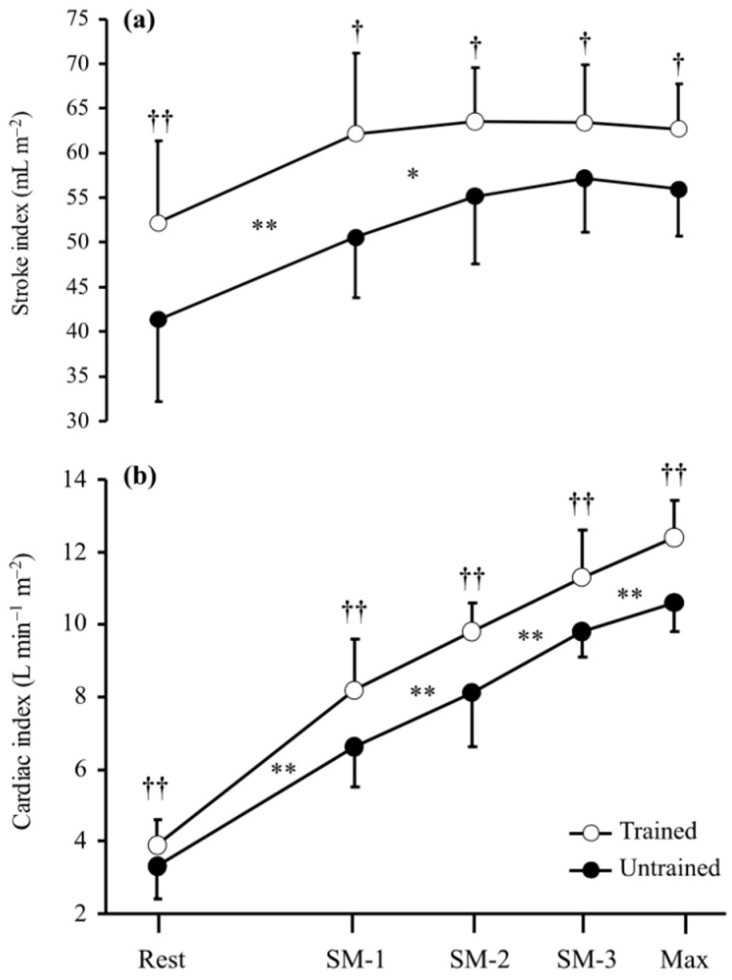
Stroke index (**a**) and cardiac index (**b**) at rest and during submaximal and maximal exercise in the child cyclists and untrained children. Difference between workloads for both groups (* *p* < 0.05 and ** *p* < 0.001). Difference between the child cyclists and the untrained children († *p* < 0.01 and †† *p* < 0.001). Nottin et al., 2002 [34]. Reprinted with permission from Nottin et al. [34], 2002, John Wiley and Sons.

Nottin et al. [34] evaluated the submaximal cardiac responses as part of an incremental protocol to exhaustion in endurance trained (2 years), pre-adolescent cyclists and age-matched, recreationally active control participants. Methodologically, stroke volume index (SVIndex) was derived from the product of the velocity time integral of (VTI) x resting aortic root area and adjusted for body surface area. Velocity time integral was obtained from the ascending aorta blood flow velocity using a Pedof (dedicated continuous wave Doppler) probe placed at the suprasternal notch. This methodological approach was adopted by all the studies [1,33] in this review that have acquired submaximal echocardiographic derived SVI, and the reproducibility of this data acquisition technique is described in Section 3. At any given absolute submaximal exercise intensity (35 W increments), the athletes presented with a higher SVIndex than the recreationally active control group. Furthermore, the pattern of change from rest throughout the submaximal exercise domain was identical between the two groups (Figure 1), a pattern also noted by Rowland et al. [35] in highly-trained adolescent cyclists. These authors concluded that the higher SVIndex in the youth athletes at any given absolute submaximal intensity was a product of increased preload as evidenced by an increase in left ventricular end-diastolic diameter (LVEDD) during exercise along with decreased afterload [decreased systemic vascular resistance (SVR)]. Limitations existed in this novel investigation; SVR was derived from estimates of cardiac output (Q) and mean blood pressure (MBP), consequently any error in these two variables would result in a mis-estimation of SVR. The consequence of the superior aerobic capacity (cyclists: 58.5 vs controls 45.9 mL/kg/min) in the trained pre-adolescent cyclists made inter-group submaximal comparisons at absolute exercise intensities challenging as each absolute intensity would represent a different relative exercise intensity for each group. Inter-group comparisons made at the same relative exercise intensity would have overcome this study design limitation.

The methodological advancements in echocardiography have allowed for a deeper interrogation of the submaximal in-exercise cardiac responses in the youth athlete and an ability to delineate the underpinning systolic and diastolic mechanisms responsible for the global (SVIndex and QIndex) cardiovascular patterns previously noted during submaximal exercise in these young athletes [34,35].

With advancements in pulsed wave and tissue Doppler technologies, Rowland et al. [23,32] evaluated the submaximal responses at absolute workloads as part of an incremental exercise protocol to exhaustion in adolescent male [23] and female [32] soccer players against age-and sex-matched controls. These studies demonstrated that there was no evidence of superior diastolic function during exercise (E, early diastolic tissue velocity (E′) and ratio of early mitral inflow velocity to early diastolic tissue velocity (E/E′)) being the primary determinants for the superior aerobic capacity seen in these elite male and female adolescent soccer players.

Work by Unnithan et al. [33] with elite male youth soccer players from two English Premier League youth soccer academies highlighted the significant increase in training volume that these young athletes were being exposed to (9.4 h per week), which were similar to that seen by Rowland et al. [23] in their study with elite male adolescent soccer players. The data confirmed the theory that at an increasingly younger chronological age, these youth athletes were being exposed to greater training loads, which heightened the need to evaluate their in-exercise cardiac performance. To evaluate the functional cardiac responses at the same metabolic load, Unnithan et al. [33] made inter-group comparisons between the SP and recreationally active controls at two relative exercise intensities (46% and 60% maximal oxygen uptake $\dot{(V}O_{2\max})$).

**Figure 2:**
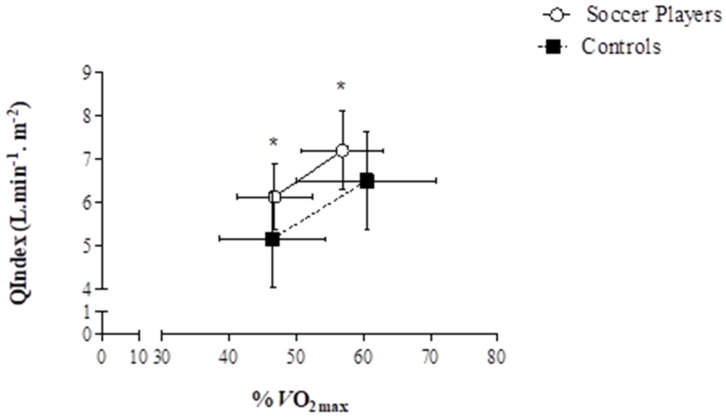
QIndex responses of soccer players (*n* = 22) and controls (*n* = 15) at the two comparable, relative exercise intensities (RE (1) and RE (2)). The symbols denote the mean values. Horizontal error bars denote the variability (SD) in relative exercise intensity (%VO_2_max) and vertical error bars denote the variability (SD) in QIndex. At both RE (1) and RE (2), the SP demonstrated significantly greater QIndex responses than the CON. * denotes *p* ≤ 0.05. Unnithan et al., 2018 [33]. Reprinted with permission from Unnithan et al. [33], 2018, John Wiley and Sons.

**Figure 3:**
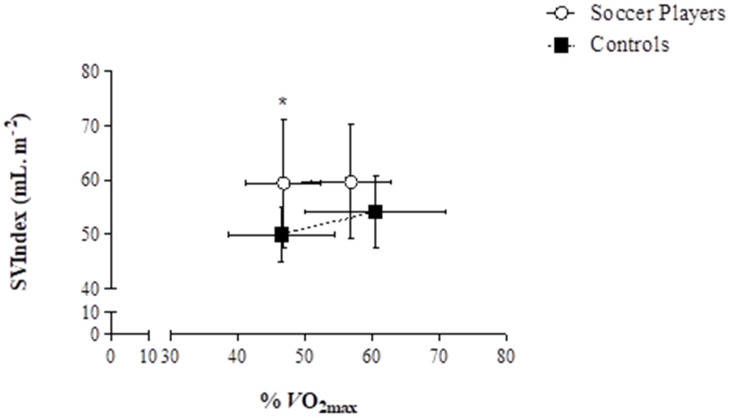
SVIndex responses of soccer players (*n* = 22) and controls (*n* = 15) at the two comparable, relative exercise intensities (RE (1) and RE (2)). The symbols denote the mean values. Horizontal error bars denote the variability (SD) in relative exercise intensity (%VO_2_max) and vertical error bars denote the variability (SD) in SVIndex. At RE (1), the SP demonstrated significantly greater SVIndex responses than the CON. There was no significant inter-group difference in SVIndex at RE (2). * denotes *p* ≤ 0.05. Unnithan et al. [33]. Reprinted with permission from Unnithan et al. [33], 2018, John Wiley and Sons.

There was evidence of greater SVIndex and QIindex in the soccer players at the same relative exercise intensity compared to controls; this confirmed the findings from Nottin et al. [34] in endurance trained pre-adolescent cyclists. Subsequently, using the same methodological approach adopted by Rowland et al. [23] (2D and tissue Doppler imaging), Unnithan et al. [33] investigated the potential underpinning systolic and diastolic mechanisms driving the superior SVIndex and QIndex noted in their study (Figure 2 and Figure 3). There was no evidence of any superior systolic function based upon the lack of differences in S′; this was corroborated by the application of STE, which also resulted in no significant differences in peak global longitudinal ε between groups. The role of peak global longitudinal ε as the major determinant of the contractility of myocardial fibres during submaximal exercise was challenged by evidence from semi-supine submaximal cycle ergometry exercise in elite adolescent soccer players [26]. These data suggested a plateauing of longitudinal ε during the transition from rest to 50 W with a concomitant increase in circumferential ε up to 150 W. This pattern is like those reported in adults with a progressive increase in LV rotations and circumferential ε, which was not observed in longitudinal ε after the initial change from rest to exercise [36]. Moreover, Pieles et al. [26] suggested that the main mechanism for contractility increases during submaximal exercise was driven by circumferential rather than longitudinal ε. However, the study did not contain a non-trained control group; thus, it is not clear how this strain pattern with incremental exercise differs to a less fit group of non-athletes. Therefore, a study to assess athlete-control differences is needed, especially since others have found reduced circumferential ε in a population of presumably non-athletic children during supine exercise at 160 bpm [27]. Still, given the methodological implications of STE at high intensities and thus, high heart rates (see Section 3), this warrants consideration and further study. Moreover, a major limitation with respect to global ε data is its inter-dependence on preload and afterload. Arguably, evaluation of systolic (SSR) and diastolic strain rate (DSR) is less dependent on load than ε [26]. Consequently, the lack of inter-group differences in SSR and DSR in the Unnithan et al. [33] study substantiated the finding of the lack of inter-group differences in peak global longitudinal ε at two submaximal, relative exercise intensities during upright cycle ergometry. Using a semi-supine cycle ergometer, Pieles et al. [29] demonstrated increases in both longitudinal and circumferential strain rate with respect to baseline up to a workload of 150 W in adolescent soccer players. The lack of control group in this study prevents these data from being contextualised in terms of both magnitude and pattern of response of strain rate.

Interrogation of diastolic function using TDI demonstrated a greater mitral E during submaximal exercise [33], and there was evidence to support the contention that determinants of preload, such as a greater left ventricular end-diastolic volume (LVEDV) at rest, were responsible for this superiority in these pre-adolescent soccer players. The greater mitral inflow velocity during submaximal exercise was also noted in adolescent male soccer players, without achieving statistical significance [23]. The lack of inter-group differences between youth soccer players and controls in E′ [23,32,33] suggested that the enhanced mitral inflow velocities were not driven by enhanced “down-stream” ventricular suction effects during submaximal exercises. Surrogate markers of ventricular filling pressures, such as E/E′, also demonstrated no significant differences during submaximal exercise in pre-adolescent [33] and male and female adolescent soccer players [23,32] and their respective age-matched controls.

The next phase of the evolution of the in-exercise echocardiographic evaluation of the highly trained pre-adolescent and adolescent athlete is to delineate the influence of growth and maturation from the impact of the training stimulus over a prolonged training exposure. The importance of this question lies in determining the magnitude of functional cardiac adaptation that results from the training stimulus at a time when youth athletes continue to be exposed to increasing training volumes and are simultaneously undergoing a period of volatile growth.

There are a paucity of studies in this area, but Unnithan et al. [1] addressed this question by exploring functional adaptations during submaximal exercise using 2D, tissue-Doppler, and cardiac ε markers across a three-year period. Elite youth soccer players from two English Premier League youth soccer academies were evaluated annually using submaximal cycle ergometry at the same relative metabolic load (45% ${\;\dot{V}O}_{2\max})$ from the ages of 11 to 14 years, as they progressed through the youth soccer academy system. The training stimulus within the club and physical activity outside the club led to a cumulative exercise load increasing from 10.5 to 13.4 h of training per week. These players were compared with a group of recreationally active age-matched peers (averaging 3.5 h per week across the three years), who were also evaluated annually at the same relative metabolic load (~45% ${\;\dot{V}O}_{2\max}$) as the soccer players over the three-year period.

After controlling for the influence of growth and maturation, QIndex during submaximal exercise increased significantly from year one to year three in the soccer players compared to the controls. There was a disproportionate increase in year two that was time-aligned with a significant increase in training volume. This increase was also directly aligned to an increase in lean body mass in the soccer players. It is possible to speculate that this increase in metabolically active tissue acted as a stimulus for the exercise-related increase in QIndex.

**Figure 4:**
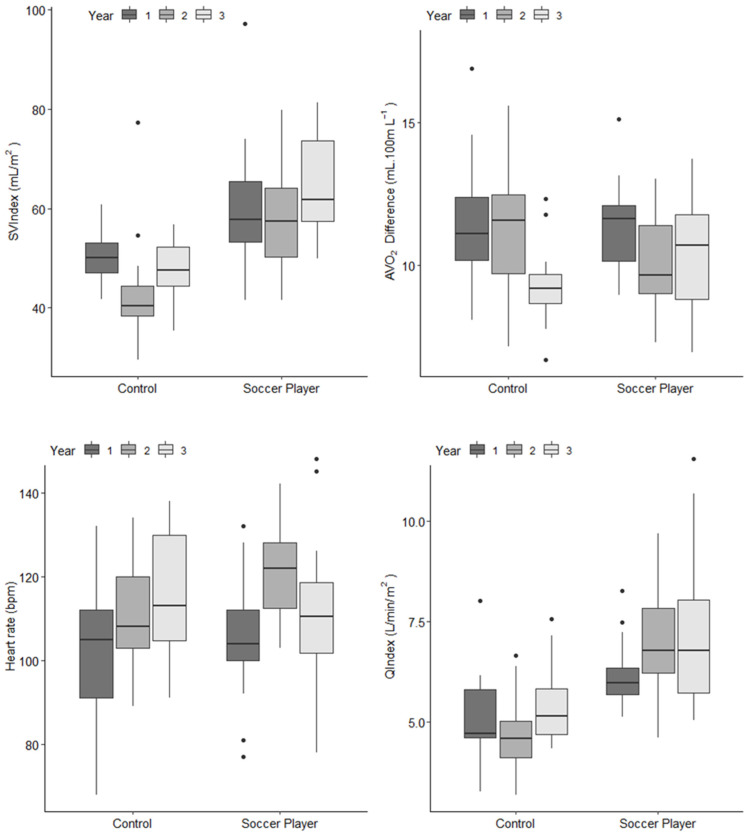
Changes in heart rate (HR), cardiac index (QIndex), stroke volume index (SVIndex), and arterial-venous oxygen difference (AVO_2_ difference) at approximately 45% $\dot{V}O_{2peak}$ in the control participants and soccer players over the course of the three-year observational study. All values are median and inter-quartile range. Unnithan et al. [1]. Reprinted with permission from Unnithan et al. [1], 2022, John Wiley and Sons.

**Figure 5:**
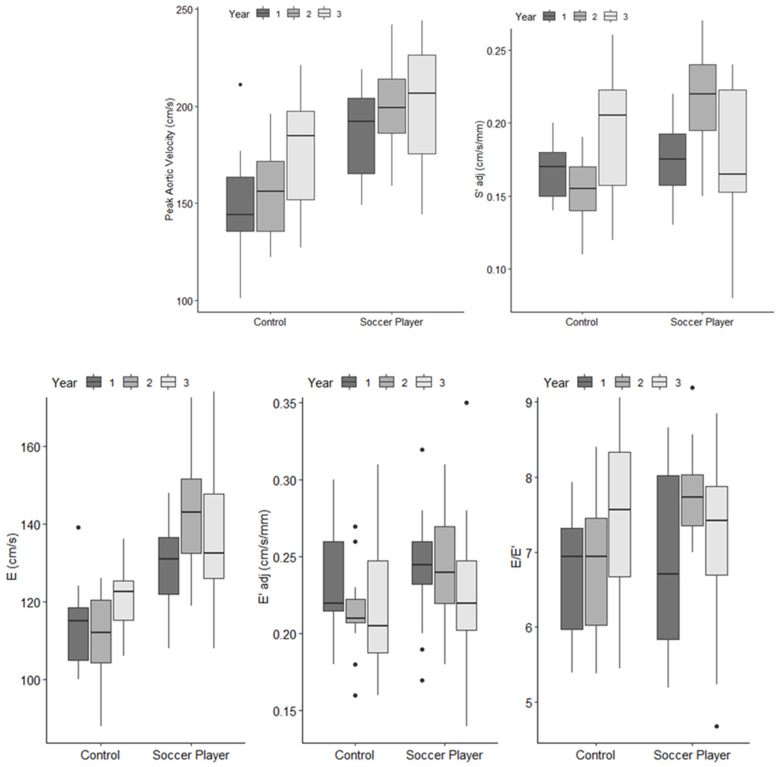
Changes in TDI derived markers of systolic (peak aortic velocity and S′adj) and diastolic function (E, E′adj and E/E′) during submaximal exercise at approximately 45% $\dot{V}O_{2peak}$ in the control participants and soccer players over the course of the three-year observational study. All values are median and inter-quartile range. Unnithan et al. 2022 [1]. Reprinted with permission from Unnithan et al. [1], 2022, John Wiley and Sons.

Furthermore, this increase in QIndex was also coupled to superior SVIndex across the three years (Figure 4). It was interesting to note that the magnitude of the SVIndex seen in the adolescent soccer players in the Unnithan et al. [1] study (64 mL/m^2^) was greater than that seen by Rowland et al. [23] in their cross-sectional study with adolescent soccer players (57 mL/m^2^). This disparity in SVIndex between the two studies could have been a product of the far greater training load that the adolescent soccer players were exposed to in the Unnithan et al. [1] study compared to the Rowland et al. [23] study and/or the comparisons being made at relative and absolute exercise intensities, respectively. Increases in SVIndex can be driven by an increase in pre-load, decrease in afterload, and/or augmented myocardial contractility. There was evidence to support the contention of preload enhancement through training via the significant increases in E and E′ across the three years after adjusting for growth and maturation (Figure 5), with particularly significant increases in temporally aligned with the significant training volume increases in year two.

## 5. Insights into the Youth Athlete’s Heart during Echocardiographic Assessment at Maximal Exercise

Greater ${\;\dot{V}O}_{2\max}$ in youth athletes could be explained through central adaptations within the paediatric athlete’s heart. Several reports have demonstrated that adolescent athletes have larger indices of SV than untrained controls, despite similar patterns of response from rest to maximal exercise [23,32,34]. Thus, a larger ${\;\dot{V}O}_{2\max}$ in paediatric athletes is likely the consequence of a superior displacement in the SVIndex curve, since peripheral extraction (arterial-venous oxygen difference [a-v O_2_) were similar at maximal exercise in trained and untrained athletes [23,32,34]. It is possible, therefore, that the higher stroke volume index at maximal exercise is facilitated by resting, structural adaptations, including greater LV dimensions and mass [3]. Beyond O_2_ delivery and extraction, [23,32] also documented similar early diastolic function was derived through mitral inflow and tissue Doppler velocities in both male and female soccer players compared to controls at maximal exercise. Comparable increases in LV systolic and diastolic function during exercise, between athletes and non-athletes, may suggest that the paediatric athletic heart is not more advantageous at maximal exercise than controls, yet baseline physiological adaptation could facilitate an upward shift in cardiac performance at maximal exercise. Nonetheless, a more recent study did observe superior systolic tissue velocity (S′) in paediatric soccer players than controls [33]. Although it remains to be elucidated how this contributes to stroke volume at maximal exercise, which did not differ between groups. Nonetheless, the paediatric athlete’s heart appears to be capable of maintaining high stroke volumes at maximal exercise despite increasing heart rates and by extension, reduced filling time [32]. Further interrogation of LV function through myocardial mechanics (LV ε, rotation and twist) could provide additional insights into cardiac performance in the paediatric athlete’s heart at maximal exercise. While echocardiographic assessment at (near)maximal intensities is undoubtably challenging during exercise stress (see Section 4) and has yet to be conducted in children, STE derived LV mechanics has been achievable at near maximal intensities (90% peak power) in adults [21].

Future improvements in technology and applications of 2D and 3D echocardiography may provide opportunities to evaluate LV function through ε imaging in adolescent athletes. In the interim, it may be appropriate to assess LV mechanics during submaximal exercise and then immediately at the cessation of maximal exercise, which has been conducted previously [37]. However, it must be acknowledged that this is unlikely to represent true maximal performance and would be dependent on the rate of recovery and/or the speed of image acquisition. Indeed, LV ε in adolescent footballers has been shown to reduce to near baseline levels, after increasing during submaximal exercise, by 2 min recovery (−2.7% ε compared with rest) [26], albeit still being statistically greater than rest even at 6 min recovery (−1.4% ε compared with rest). Thus, there may be a window of opportunity for assessment, but the differentiation between active and passive elevations in LV ε when compared to baseline may be indistinguishable.

## 6. Future Directions

Methodologically, the modes of evaluation for the in-exercise echocardiographic evaluation of the highly trained, elite youth athlete have been the upright cycle ergometer and the semi-supine ergometer. There needs to be a technique developed that allows these data to be collected using the treadmill, to continue to refine the techniques required to capture more upright in-exercise echocardiographic data. The main rationale is that other than for trained youth cyclists, the endpoint for most other youth athletes will be local muscle fatigue rather than central fatigue during an incremental cycle ergometer test to exhaustion. Furthermore, body position and crank length can also alter the haemodynamic response to submaximal [31] and maximal exercise, issues that would be obviated with treadmill exercise. Irrespective of the mode of exercise, there needs to be far more global cardiac, tissue-doppler, and particularly ε data collected using echocardiography during exercise to generate normative reference values against which other youth populations (healthy and diseased) can be contextualised. With the significant increase in elite, female youth athletes and the associated high volume of training in these individuals, there is an urgent need for more in-exercise studies to elucidate left ventricular responses at submaximal and maximal exercise intensities. Similarly, there is a paucity of in-exercise data relating to youth athletes of different ethnicities.

In particular, assessment during exercise, albeit with suitable image quality derived from STE, is required to determine the physiological benefit of resting myocardial adaptations to both structure and function in youth athletes. There is a need for more well-designed, true longitudinal studies in-exercise echocardiographic studies to complement the existing work in this area [1]. Moreover, studies are required to evaluate RV function during exercise as opposed to solely LV function. While some have recently studied RV function at rest in youth soccer players [10], these data warrant further study during exercise, as there exist limited data in this area [38].

While this review focused on the use of echocardiography, cardiac magnetic resonance (CMR), an alternative modality, warrants mention and further exploration. LV and RV structures have been documented in paediatric athletes using CMR [39], which may present some advantages to 2D echocardiography, owing to greater spatial resolutions [9]. LV and RV mechanics have also been assessed in pre-adolescent soccer players [40], and with good inter-modality correlation between CMR feature tracking and STE [41]. Nevertheless, it may not be feasible to use modalities interchangeably due to underestimation in the former [41], and differences in values of the athlete’s heart structure derived from echocardiography and CMR [9]. Underestimation of LV mechanics with CMR could be related to temporal resolution [41], which may become more problematic during exercise with high heart rates, particularly when phases of the cardiac cycle, such as isovolumic contraction and relaxation, shorten. Indeed, temporal as opposed to spatial resolution impacts strain and strain rate values [42].

## 7. Summary

The youth athlete’s heart is associated with myocardial adaptations at rest to both structure and function. Based on multiple lines of study, these physiological adaptations may facilitate an upward shift in LV output (SV and Q) during submaximal and maximal exercise, which could, in turn, contribute to greater aerobic capacities of young athletes. Furthermore, the pattern of in-exercise responses is similar, irrespective of sports discipline, and based on these data there also appears to be no maturational threshold for cardiac adaptation to training in these youth athletes.

Technological advancements have enabled assessment of more contemporary markers of LV function through STE, however, more studies are needed to compare youth athletes and controls during exercise. With in-exercise assessment of cardiac function, there are several methodological considerations required, including appropriate utilisation of technologies, physiological significance related to a given sport, body position during acquisition, and offline analysis of STE derived data.
